# Contrast enhanced CT and MRI interchangeably reflect tumor characteristics in murine pancreatic cancer

**DOI:** 10.1038/s41598-026-56008-4

**Published:** 2026-06-05

**Authors:** Lea Würfel, Peter Niehaus, Geoffrey J. Topping, Mariia Semina, Markus Mittelhäuser, Susanne Kossatz, Marina Lesina, Fabian Lohöfer, Franz Schilling, Uwe Karst, Rickmer Braren, Irina Heid

**Affiliations:** 1 Institute of Diagnostic and Interventional Radiology, TUM School of Medicine and Health, TUM University Hospital Rechts der Isar, Technical University of Munich, Munich, Germany; 2 Institute of Inorganic and Analytical Chemistry, University of Muenster, Muenster, Germany; 3 Department of Nuclear Medicine, TUM School of Medicine and Health, TUM University Hospital Rechts der Isar, Technical University of Munich, Munich, Germany; 4 German Cancer Consortium (DKTK) partner site Munich, Munich, Germany; 5 Institute for Tumor metabolism, Comprehensive Cancer Center Munich TUM, TUM School of Medicine and Health, TUM University Hospital Rechts der Isar, Technical University of Munich, Munich, Germany; 6 Department of Diagnostic and Interventional Radiology and Nuclear Medicine, University Medical Center Hamburg-Eppendorf, Hamburg, Germany

**Keywords:** PDAC, Iodine, Gadolinium, DCE-MRI, CE-CT, LA-ICP-MS, Cancer, Medical research, Oncology

## Abstract

Contrast-enhanced computed tomography (CE-CT) and dynamic contrast-enhanced magnetic resonance imaging (DCE-MRI) are commonly used for tissue characterization, with CE-CT typically preferred in human studies and DCE-MRI in murine models. This raises the question of comparability of intra-and cross-species study results, as techniques differ and signal intensities following contrast agent (CA) administration reflect a complex interplay of systemic physiology and local tissue characteristics. This pilot study compares in vivo mean values of regions of interest (ROIs) obtained from CE-µCT (NanoScan^®^ SPECT/CT) and 7 T DCE-MRI (Agilent Discovery MR901 magnet with Bruker AVANCE III HD electronics) and evaluates ex vivo CA distribution using laser ablation–inductively coupled plasma–mass spectrometry (LA-ICP-MSI). A genetically engineered mouse model of pancreatic ductal adenocarcinoma (PDAC) served as the experimental system. Image-derived regional mean Hounsfield unit (HU) and area under the curve at 60 s (AUC₆₀) values were correlated with mean local iodine (iomeprol) and gadolinium (gadopentetate dimeglumine) concentrations. Semi-quantitative analysis of HU and AUC₆₀ enabled excellent distinction of histologically defined tumor regions with low versus high tumor cellularity (*p* < 0.0001 for both). A strong intermodal correlation was observed between regional HU and AUC₆₀ values (*r* = 0.91, 95% CI = 0.78–0.97), as well as between iodine and gadolinium ion concentrations (*r* = 0.86, 95% CI = 0.55–0.96). These findings demonstrate that CE‑µCT and DCE‑MRI show comparable trends as prognostic imaging biomarkers of tumor cellularity in murine PDAC, underscoring their complementary value for cross‑species translational imaging research.

**Supplementary Information:**

The online version contains supplementary material available at 10.1038/s41598-026-56008-4.

## Introduction

Computed tomography (CT) and magnetic resonance imaging (MRI) are commonly used radiological imaging techniques for detection, classification and therapy response monitoring of solid tumors, such as pancreatic ductal adenocarcinoma (PDAC). Static and dynamic contrast-enhanced (CE)-CT and dynamic contrast-enhanced (DCE)-MRI provide information related to vascular properties (e.g. tissue vascularity, perfusion and vessel wall permeability or leakiness)^1, 2^, tissue composition (e.g. stroma content, tumor cellularity or tumor aggressiveness)^3, 4, 5, 6^ and treatment effects (e.g. phenotype-specific perfusion changes or status of the tumor margin)^6, 7^.

CE-CT detects enhanced X-ray attenuation upon injection of iodinated (I) contrast agents (CA) such as iomeprol^8^. Observed regional signal attenuation is the result of a complex interplay of local (i.e. tissue composition) and systemic (i.e. cardiovascular, renal) properties. After intravenous administration and vascular distribution, iomeprol enters the extravascular, extracellular fluid space via diffusion, with a mean distribution half-life of 27 min^9^ and an elimination half-life of 109 min in humans^10, 11^. Iomeprol is a non-ionic tri-iodinated monomer with a molecular weight of 777.1 g/mol^9^ that shows no binding to plasma proteins, and is excreted unmetabolized through the kidneys^11, 12^. It is characterized by low allogenic potential and a favourable pharmacodynamic profile due to its low osmolality, low viscosity, and high water solubility^11^.

MRI uses strong magnets and radiofrequency oscillating electromagnetic fields to create detailed images without the need for ionizing radiation and is especially suitable for repeated high contrast imaging of soft tissues. In MRI, Gadolinium (Gd)-based chelate complexes of DTPA (diethylenetriamine pentaacetate) such as gadopentetate dimeglumine are used as exogenous CA. CA arrival and distribution in tissues is commonly recorded dynamically^6, 13^. Paramagnetic Gd shortens the spin-lattice relaxation time T1 of water protons in tissues, enhancing signal intensity in T1-weighted MRI sequences. In addition to the above described local and systemic effects on the signal, additional technical details (flow and concentration-dependent signal effects) must be taken into consideration in signal interpretation. Gadopentetate dimeglumine is classified as an acyclic, ionic CA with hydrophilic properties and a molecular weight of 938 g/mol^13^. The distribution and renal elimination half-lifes reported for gadopentetate dimeglumine in humans are 12 and 90 min^14^. Similar to iomeprol, gadopentetate dimeglumine passively distributes in the extravascular and extracellular fluid space via diffusion, and shows no significant interaction with plasma proteins such as albumin^15, 16^.

Because of cost and time effectiveness in comparison to MRI, CE-CT remains the primary tool for routine diagnostics^17, 18, 19^. In preclinical animal studies, however, CE-µCT is rarely applied due to its generally inferior soft tissue contrast and need for high CA concentrations (> 1 g/kg body weight)^20^, long acquisition times of several minutes and high exposure to radiation^21^. These limitations stem from slower acquisition speeds compared to the high heart rate of mice, underscoring key differences from clinical imaging in humans. Moreover, the temporal resolution of most preclinical µCT protocols is inadequate for meaningful dynamic CE-µCT analysis. Extended acquisition times are required to achieve sufficient soft tissue contrast for abdominal anatomical localization in mice, but this precludes true dynamic characterization.

In contrast, DCE-MRI with a high temporal resolution of a few seconds allows the detection of inflow kinetics as an additional vascular parameter^22^. Moreover, DCE-MRI was shown to be well tolerated by animals in repeated measurements, and sequential DCE-MRI studies had no impact on the tumor proliferation or vascularity^21, 23^. In contrast, repeated CE-µCT measurements decreased tumor proliferation and increased tumor perfusion in a breast cancer model^21^. However, using DCE-MRI in animals and CE-CT in humans raises concerns about the clinical translatability of preclinical results, such as for example the assessment of tumor perfusion as a reflection of tumor cellularity. Therefore, comparative studies of CE-µCT and DCE-MRI with regard to in vivo imaging biomarkers and ex vivo CA distribution are needed.

Pancreatic ductal adenocarcinoma (PDAC) is characterized by major inter- and intratumoral heterogeneity in its growth pattern in mouse and human^3, 7, 24, 25^. A hallmark of PDAC is its generally poor perfusion compared to the surrounding normal pancreatic tissue due to low angiogenic potential, high interstitial pressure and collapsing vessels^26, 27^. Hounsfield Units Ratio (HUr), derived from CE-µCT, and Area Under the CA Curve of 60 s (AUC₆₀), derived from DCE-MRI, have been used to depict heterogeneity, distinguishing for example regions of high (> 40% of tumor cells, PDAC^high^) and low (< 40% of tumor cells, PDAC^low^) tumor cellularity in human and mouse^5, 6, 25^. Detecting high tumor cellularity in PDAC is clinically crucial, as it has been shown in large surgical cohorts to be a prognostic indicator of poorer survival^4, 25^. The proposed classification is based on the observation that highly cellular tumor regions exhibit fewer functional vessels compared with regions of low tumor cellularity. Consequently, these regions can be detected during the early phases of contrast agent inflow on contrast-enhanced CT and MRI^6^.

Laser Ablation-Inductively Coupled Plasma-Mass Spectrometry (LA-ICP-MS) can be applied to analyze CA distribution patterns ex vivo. It has been used to study specific distribution of elements like Gd and I^28, 29^. Previous studies have shown consistency in the distribution of Gd in vivo (MRI) and ex vivo (LA-ICP-MS) in two implanted murine breast cancer models and porcine brain tissue^30, 31^.

The aim of the pilot study was to quantify and correlate semi-quantitative in vivo imaging biomarkers - such as region of interest (ROI) mean values of reconstructed, normalized, and time-averaged image intensities in HU images or AUC₆₀ maps - with mean ex vivo CA tissue concentrations at 60–63 s after CA administrations. We focused on AUC₆₀ because a closely related metric, the initial AUC₉₀ (iAUC₉₀), has been reported to correlate not only with CE-µCT-derived structural parameters - such as vessel volume, branch density, and length - but also with the functional DCE-MRI parameter K^trans^ in preclinical models^32^. Pharmacokinetic modeling (PKM) of functional parameters such as (K^trans^), or reverse volume transfer coefficient (K_ep_) can provide quantitative information of tumor perfusion and microvascular function, as well as detect changes in tumor microenvironment^33^. However, PKM is complex and error-prone measurement because it is highly dependent on arterial input function, which is intra-individually heterogeneous and challenging to determine in small animals^34^. A model-free, robust surrogate AUC_60_ can also reflect perfusion, vessel density, and microvascular functionality^6, 32, 35^. Therefore, we performed iomeprol-enhanced CE-µCT and gadopentetate dimeglumine-enhanced DCE-MRI in the same PDAC bearing mice, followed by ex vivo LA-ICP-MS of I and Gd concentrations and regional analysis of co-registered data.

## Materials and methods

### Animal handling and anesthesia

All experiments were approved by and performed according to the guidelines of the local animal protection and welfare review board (Regierung von Oberbayern, Munich, Germany, Approval Number ROB-55.2- 2532.Vet_02-18-92). The approval was obtained before study initiation, and the results are reported following the ARRIVE guidelines. All mice were maintained on a standard light-dark cycle with food and water. Mice were anesthetized with isoflurane 1.5-2% (v/v) in an oxygen flow rate of 2 L/min. Breathing rates (50–70 bpm) and temperature (37–39 °C) were monitored. As described below, CAs were injected intravenously (i.v.) via a tail vein catheter. All animals were euthanized using cervical dislocation under deep anesthesia of 5% isoflurane and 2 L/min 100% O_2_.

### Study subjects and tissue preparation

Genetically engineered mice of the *Ptft1a*^*wt/cre*^*KRAS*^*wt/G12D*^*TP53*^*fl/fl*^ *(KPC)* genotype (*n* = 3) were used in this study. These animals develop several histopathologically different tumor nodules within the pancreas of one animal and have been well characterized in previous studies^6, 25^. One tumor-bearing mouse with a very heterogeneous tumor containing numerous histologically and radiologically well-defined ROIs (animal no. 1, Suppl. Figure 1) was used for the in vivo comparison of CE-µCT and DCE-MRI. This animal was subjected to two CE-µCT scans, before and directly after an injection of iomeprol (Imeron400^®^, 1 g/kg, Bracco, Frankfurt, Germany) dissolved in 0.9% NaCl. We administered 1 g iodine per kg body weight, corresponding to approximately twice the human‑equivalent dose, to enhance contrast while minimizing the risk of adverse effects associated with iodine accumulation in mice, as previously reported^20^. Two days later, the same animal was subjected to DCE-MRI with a bolus dose of gadopentetate dimeglumine (Magnograf^®^, 0.5 mmol/kg, Jenapharm GmbH, Jena, Germany) dissolved in 0.9% NaCl, and subsequently sacrificed. Pancreatic tumor tissue was sliced with a scalpel in 6 axial slices (3–5 mm thick) and snap-frozen on dry ice in axial orientation to enable co-registration with imaging. The tissue of this animal was only partially included in further studies with LA-ICP-MS (Suppl. Figure 2) due to absence of CA clearance period after the DCE-MRI.

Two further tumor-bearing animals (no. 2 and 3) with heterogeneous tumor regions measuring 2.5–6 mm in diameter were subjected to DCE-MRI with a bolus dose of 0.5 mmol/kg gadopentetate dimeglumine followed by a CA clearance period of two days. Subsequently, animals were injected with a combination of iomeprol (500 mg/kg, approximating the human-equivalent dose) and 0.5 mmol/kg gadopentetate dimeglumine, both diluted in 0.9% NaCl, to ensure their simultaneous in vivo circulation. The combined use of gadolinium‑based and iodinated contrast agents has been reported as safe^36^ and their administration on the same day is clinically acceptable^37^. Furthermore, recent data indicate that the early biodistribution of these agents is primarily determined by blood flow and vascular permeability, with minimal interference from co‑administration during initial time points^38^. The CAs were allowed to distribute for 60 s within the murine body, followed by rapid euthanasia through cervical dislocation in deep anesthesia, tissue sample extraction and snap freezing on dry ice. All tumors were sliced with a scalpel in 4–6 axial slices (3–5 mm thick) and frozen in axial orientation to enable co-registration with imaging. Frozen samples were embedded in the same axial position into a hydrogel of 7.5% HPMC/2.5% PVP as previously described^39^, cryosectioned to 10 μm thickness and mounted onto Superfrost glass slides for LA-ICP-MS. Consecutive slices were stained with Mayer’s hematoxylin and eosin (H&E) for manual co-registration with imaging. Tumors were manually defined as PDAC^high^ and PDAC^low^ based on previously set classification, with PDAC^high^ regions containing > 40% tumor cells and PDAC^low^ regions containing < 40% tumor cells^6^. All contrast agent injections were performed manually by an experienced operator to ensure consistency; however, minor variability in bolus injection rate or timing could not be entirely excluded and may contribute to inter- and intra-animal variability in AUC₆₀ and HU measurements.

### Imaging protocol and data analysis

For MRI, a 7 T preclinical scanner (Discovery MR901 magnet and gradient system, Agilent, Santa Clara, CA, USA; AVANCE III HD electronics, Bruker, Billerica, MA, USA) with a 31 mm ^1^H/^13^C dual-tuned volume resonator (RAPID Biomedical, Rimpar, Germany) was used. For each animal, an axial T2-weighted anatomical MRI (T2w rapid acquisition with relaxation enhancement (RARE), 10 slices, scan duration 4 min, 0.175 × 0.175 × 1.75 mm^3^, echo time TE = 48 ms, repetition time TR = 6000 s) and corresponding axial DCE-MRI (T1w spoiled gradient-recalled echo (FLASH), 8 slices, reconstruction matrix 48 × 36, acquisition matrix 33 × 30 with 1.22 phase-encode interpolation and 1.45 partial Fourier frequency encoding, voxel size 0.583 × 0.583 × 1.75 mm^3^, temporal resolution 0.6 s, total scan duration 5 min, TE = 0.933 ms, TR = 20 ms, flip angle = 30°) were performed. These settings were selected as the time-dependent CA distribution curves in murine PDAC matched previously observed patterns from our studies on 1.5 T clinical scanner^6^. DCE-MRI were analyzed using in-house software (Matlab, Version R2024b). ROIs drawn on the T2w images were automatically transferred to the corresponding AUC₆₀ maps. For each voxel, after normalizing to the pre-contrast signal average, the area under the signal curve at 60s (AUC₆₀) after bolus arrival was calculated for tumor and muscle separately. Thereafter, all consecutive histopathological sections from each mouse were correlated with the imaging data, and the tumors were classified into PDAC^low^ and PDAC^high^ as previously described^6^.

The µCT was performed on the NanoScan^®^ SPECT/CT (Mediso, Budapest, Hungary) with the following parameters: 50 kV, 600 µA, 480 projections, 300 ms each, total single scan duration 63 s. In total two scans, one native scan and one CE-scan performed directly after the intravenous application of iomeprol, were used in this study. These settings were chosen as they revealed good soft tissue contrast in the abdomen, particularly in the kidneys. µCT images, corresponding T2-weighted MRI anatomy and AUC₆₀ maps were analyzed using Horos v3.3.6 (https://horosproject.org/). For CE-µCT vs. DCE-MRI mean ROI value correlation, 18 ROIs were visually defined in animal no. 1 based on T2-weighted anatomical images (coronal and axial; Suppl. Figure 1). These ROIs were manually transferred to the corresponding regions on axial CE-µCT images and AUC₆₀ maps (previously calculated in MATLAB and imported as DICOM datasets into Horos), where mean regional Hounsfield unit (HU) and AUC₆₀ values were extracted. For this analysis, CT data were compressed to eight slices with a slice thickness of 1.75 mm within the DCE-MRI coverage using 3D multiplanar reformation (3D MPR). Finally, tumor cellularity classification was applied using corresponding histological sections. To minimize subjectivity, ROI definition and verification were performed by consensus between a clinical radiologist and a preclinical imaging specialist, using consistent anatomical landmarks and standardized viewing settings in Horos.

### Bioimaging and quantification via LA-ICP-MS

Elemental maps of the tissue thin sections were recorded using a laser ablation system model ImageBio266 (Elemental Scientific Lasers, Bozeman, MT, USA) coupled to a triple quadrupole-based ICP-MS (iCap TQ, Thermo Scientific, Bremen, Germany). Ablation of the sample was carried out at a spot size of 25 μm. The triple quadrupole was operated in oxygen mode. I and Gd were determined as ^127^I^+^ and ^158^Gd^16^O^+^respectively. For quantification of Gd and I, aqueous solutions in a concentration range of 5–500 µg g^− 1^ and 5-20000 µg g^− 1^ were produced using GdCl_3_∙6H_2_O (ThermoFisher, Kandel, Germany) and aqueous iomeprol stock solution (Imeron400^®^, Bracco, Milan, Italy). With these, matrix matched gelatin (Karl Roth, Karlsruhe, Germany) standard sections were produced in accordance with a protocol described elsewhere^40^. Gelatin standards were analyzed at identical analytical conditions for quantification. Data evaluation was carried out using the in-house developed software imajar^41^. Tumors were histologically correlated and manually co-registered with in vivo imaging.

### Statistical analysis

Statistical analysis was performed using GraphPad prism version 10. Groups were compared using unpaired student’s t-test. Mean values and standard deviations are shown for group comparisons. Pearson’s test was applied for data correlation. The confidence interval amounted to 95%. For the significance level, 5% was set so that a p-value of < 0.05 could be considered statistically significant.

## Results

### Inverse correlation between Gd-based CA accumulation and tumor cellularity in murine PDAC

**Fig. 1:**
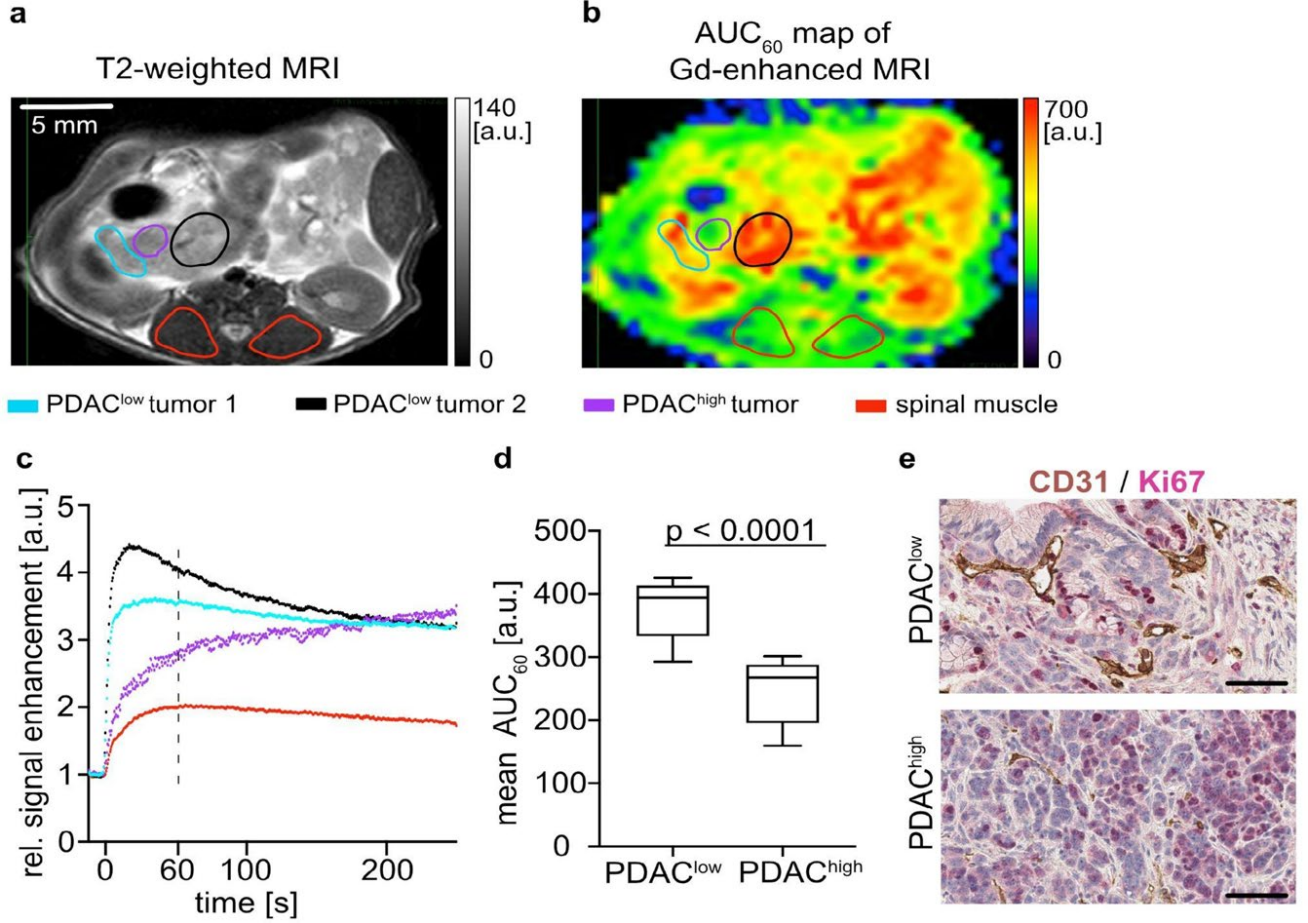
In vivo distinction of PDAC subgroups for tumor stratification. T2w image **(a)** and AUC₆₀ map **(b)** showing two well-perfused PDAC^low^ tumors (blue and black ROI) and one poorly-perfused PDAC^high^ tumor (purple circle), which were identified based on corresponding histopathological tissue samples (spinal muscles, red circles) within the animal no. 2. **(c)** Corresponding color-coded time curves of Gd-enhancement normalized to the pre-contrast signal in tumors and muscle. **(d)** Group comparison of regional AUC₆₀ values between PDAC^low^ (*n* = 12) and PDAC^high^ (*n* = 5) tumor lesions. **(e)** Double immunostaining for CD31 (brown) and Ki67 (red) depicts representative differences in vascularity and proliferative activity between murine PDAC^low^ and PDAC^high^ tumors. Scale bar = 50 μm. The sample preparation and staining protocol has been described previously^6^.

For this study, DCE-MRI with gadopentetate dimeglumine was implemented on a preclinical 7 T Bruker scanner using the same heterogeneous KPC model and same CA as in our previously published study on a 1.5 T clinical scanner^6^. As expected, DCE-MRI revealed major differences in regional AUC₆₀ values and CA uptake dynamics of PDAC in KPC mice (Fig. 1a-c). According to established histopathological classification in human and murine PDAC samples^6, 25^, two groups with different tumor cellularity (PDAC^high^ with > 40% and PDAC^low^ < 40% of tumor cells within the ROI)^6^ were defined using all available H&E slices in all three animals resulting in 12 PDAC^low^ and 5 PDAC^high^ lesions (Fig. 1d). Specifically, in vivo CA uptake was slower and exhibited a lower peak value in PDAC^high^ compared to PDAC^low^ tumors. As expected, AUC₆₀ values were significantly higher in PDAC^low^ (meanAUC₆₀ = 378 ± 44) compared to PDAC^high^ lesions (meanAUC₆₀ = 247 ± 55, *p* < 0.0001, Fig. 1d), confirming the previously reported inverse relationship between tumor cellularity and CA uptake detected in DCE-MRI^6^. Figure 1e shows immunohistological double staining for the vascular marker CD31 and the proliferation marker Ki67 illustrating representative differences in vascularity and tumor aggressiveness between murine PDAC^low^ and PDAC^high^ tumors. As previously described^6, 25^, PDAC^low^ tumors exhibit fewer proliferating cells (red nuclei) and a higher proportion of open, perfused vessels (brown staining and white lumen), whereas PDAC^high^ tumors display abundant tumor cell proliferation and a predominantly closed, dysfunctional vascular network (Fig. 1e).

### In vivo correlation of regional CE-µCT and DCE-MRI contrast behaviour in murine PDAC tumors

**Fig. 2:**
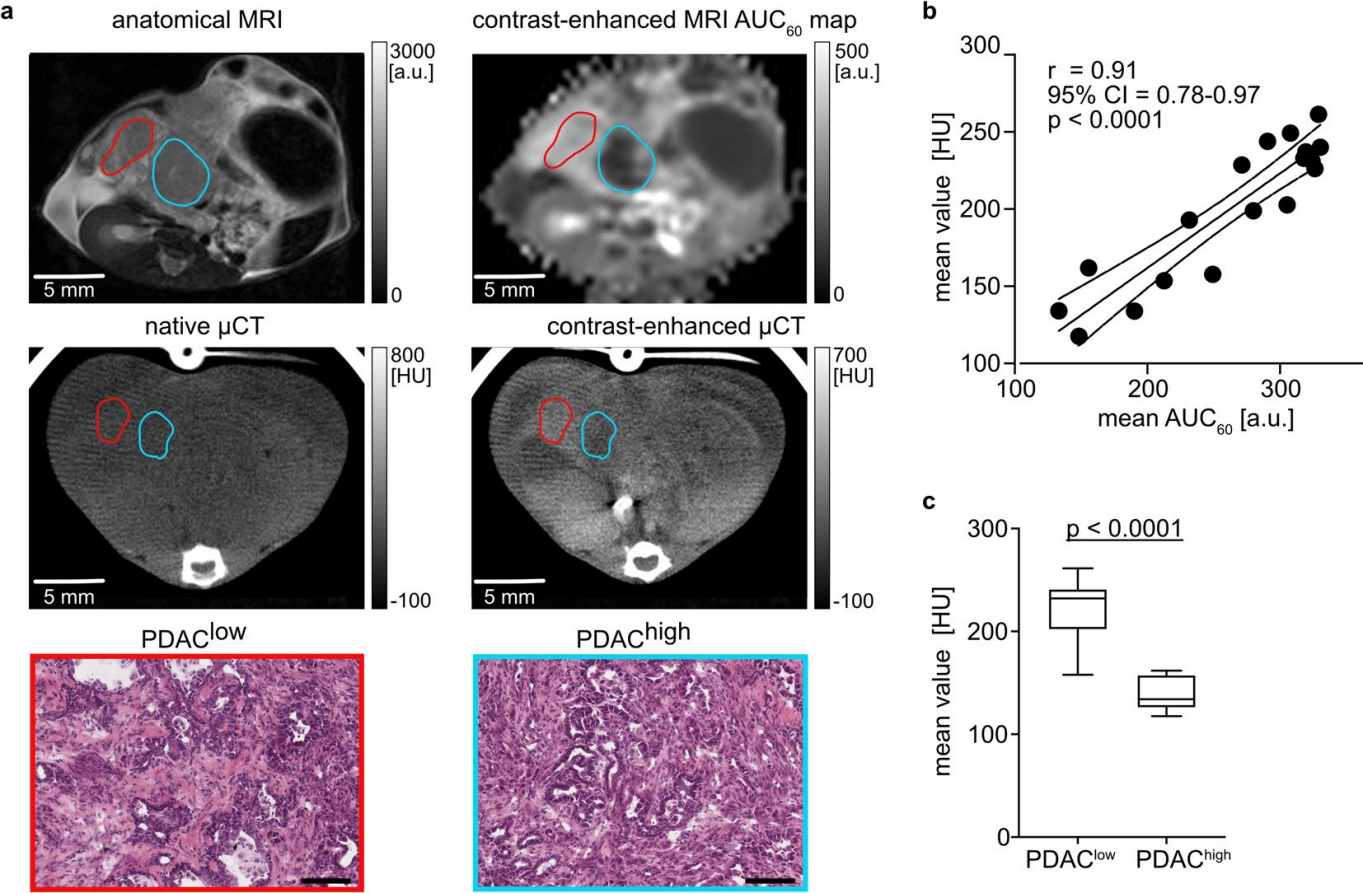
In vivo correlation of CE-µCT and DCE-MRI. (**a**) T2w image (top, left) and AUC₆₀ map (top, right) with corresponding native µCT (middle, left) and CE-µCT (middle, right) images. Circled are two distinct tumor regions (red and blue ROI) of different signal intensity and PDAC^low^/PDAC^high^ histology (bottom). For an ex vivo presentation of these tumors, please see Suppl. Figure 2. (**b**) Correlation of regional, slice-based DCE-MRI-derived AUC₆₀ values with CE-µCT-derived HU values across eighteen PDAC regions within a single animal. Regression line, Pearson correlation coefficient r, 95% confidence interval (CI) and p values are shown. (**c**) Box-and-whisker plot comparing mean HU values of different tumor cellularity groups (PDAC^low^ *n* = 13 and PDAC^high^ *n* = 5).

For a direct in vivo comparison of tissue properties between iomeprol and gadopentetate dimeglumine, a single tumor-bearing animal with multiple histologically heterogeneous lesions was first imaged using CE-µCT, followed by a two-day wash-out period and subsequent DCE-MRI. We specifically selected a single mouse with multiple heterogeneous lesions (animal no. 1) and performed a slice-based ROI analysis to ensure the most accurate assessment of perfusion-sensitive modalities. This approach minimizes confounding factors such as tumor-specific vascular variability, inter-individual differences in heart rate and contrast-agent clearance, body weight variations, partial volume effects and other systemic or technical inconsistencies. Figure 2 shows representative CE-µCT and DCE-MRI images. As expected, iomeprol CE-µCT images obtained within the first 63 s after CA injection revealed poor soft tissue contrast and showed no reliable distinction between different organs. However, visual co-registration with T2w imaging enabled tumor identification (Suppl. Figure 1, Suppl. Figure 2). Subsequent comparative analysis of tumor subregions revealed strong and significant regional correlation between mean regional CE-µCT HU and DCE-MRI AUC₆₀ values (*r* = 0.91, 95% CI:0.78–0.97, *p* < 0.0001, Fig. 2b). Moreover, a group-wise comparison of regional HU values showed significant differences between PDAC^low^ (meanHU = 224 ±27) and PDAC^high^ (meanHU = 140 ± 18) lesions (*p* < 0.0001, Fig. 2c). These results indicate that mean regional tumor values derived from both CE-µCT and DCE-MRI may be used for the in vivo distinction of tumor subregions, exhibiting differences in tumor cellularity. However, low soft tissue contrast presents a major limitation of visual interpretation of iomeprol CE-µCT. Therefore, in contrast to clinical practice, preclinical contrast-enhanced µCT alone is currently insufficient for reliable anatomical assessment and still requires complementary imaging such as T2-weighted MRI; however, emerging developments in CT technology - such as shorter acquisition times and new contrast agents optimized for preclinical soft-tissue contrast enhancement - may enable greater use of CE-µCT in the future.

### Ex vivo correlation of regional I- and Gd-concentrations in murine PDAC tumors

**Fig. 3:**
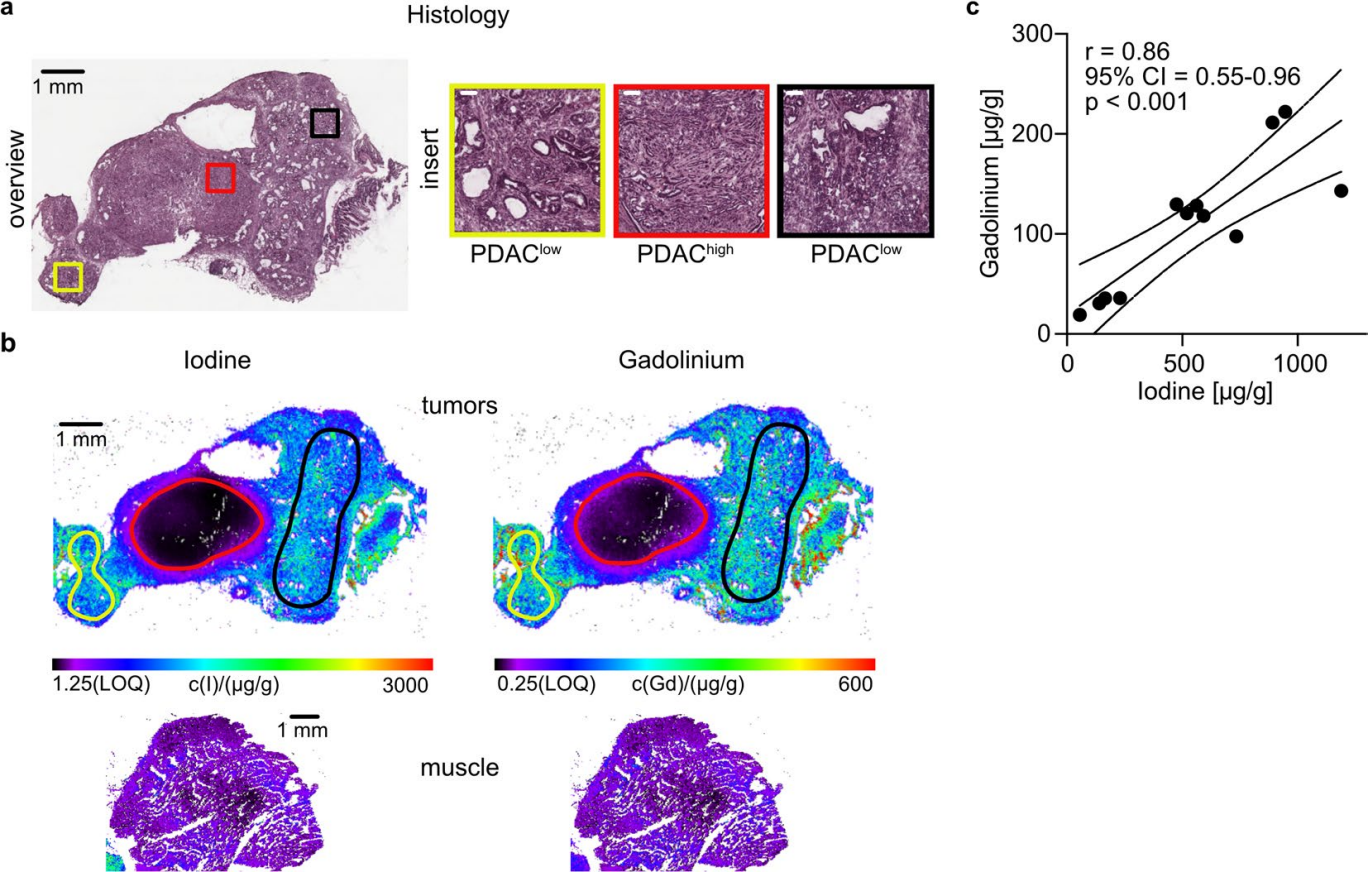
Ex vivo quantification of contrast agents uptake in PDAC subgroups by mass spectrometry imaging. **(a)** H&E-stained overview and regional magnifications (scale bar 100 μm) of stroma rich, tumor cellularity low PDAC^low^ (yellow and black ROI) and stroma poor, tumor cellularity high PDAC^high^ (red ROI) subregions within the animal no. 2. **(b)** Ex vivo LA-ICP-MS derived elemental distribution maps of I and Gd corresponding to photomicrographs in **(a)** and spinal muscle tissue. **(c)** Correlation of mean regional concentrations of I and Gd in tumors and spinal muscles. Regression lines, Pearson correlation coefficient r, confidence interval (CI) and p-value are shown.

To investigate the tissue distribution of iomeprol and gadopentetate dimeglumine in murine PDAC, mean elemental tissue concentrations of I and Gd were measured using LA-ICP-MS. A distinct, yet remarkably similar accumulation pattern of I and Gd were found within analyzed tissues as shown in Fig. 3 and Suppl. Figure 2. The PDAC^low^ tumor regions (yellow and black ROI) clearly reveal higher concentrations of I and Gd on the elemental maps in comparison to the PDAC^high^ tumor (red ROI) (Fig. 3a, b). Regional analysis showed a strong correlation between I and Gd concentrations in all analyzed tissues (*r* = 0.86; 95% CI = 0.55–0.96; *p* = 0.0004, Fig. 3c), confirming a similar distribution of the two CAs.

### Interchangeability of DCE-MRI derived AUC₆₀ imaging biomarker

**Fig. 4:**
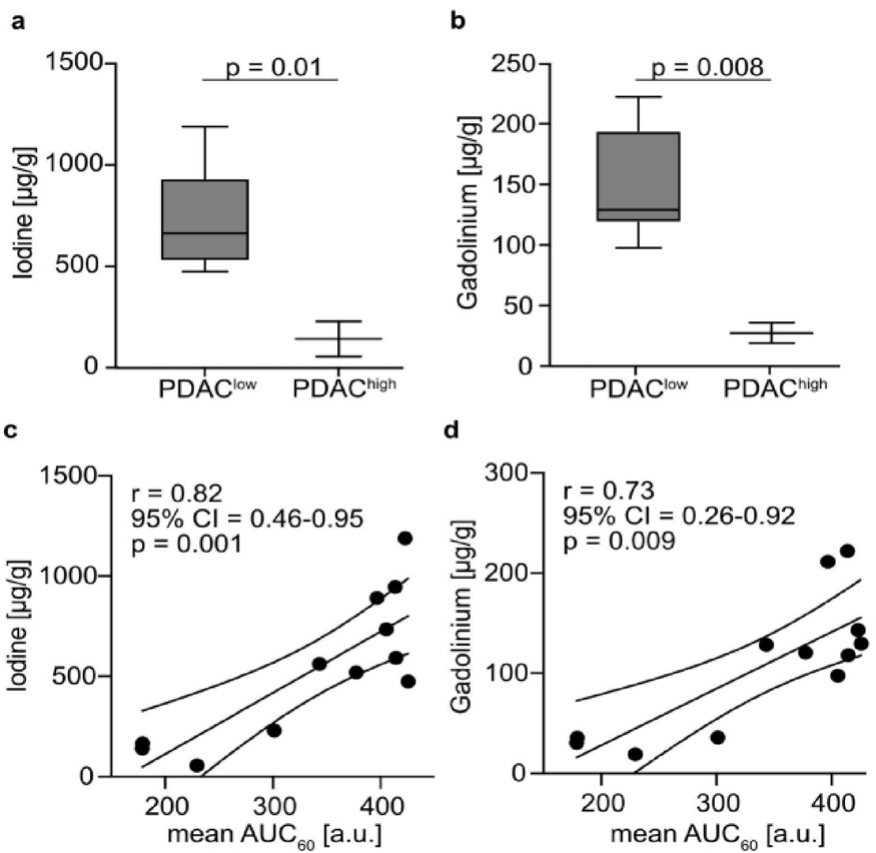
Correlation of in vivo imaging biomarker AUC₆₀ with ex vivo CA concentrations. Box-and-whisker plots showing the distribution of mean regional values of ex vivo LA-ICP-MS-derived tissue concentrations of I (**a**) and Gd (**b**) in histologically defined PDAC^low^ and PDAC^high^ subregions. Correlation of in vivo DCE-MRI-derived AUC₆₀ values with ex vivo LA-ICP-MS-derived tissue concentrations of I (**c**) and Gd (**d**).

We also found a strong correlation between CA uptake of both CAs and histopathological tumor phenotype in LA-ICP measurements (Fig. 4a and b and Suppl. Figure 2). Low concentrations of I were indicative of PDAC^high^ tumors (PDAC^high^: meanC_I _= 143 ± 123 µg/g), whereas high concentrations of I were detected in PDAC^low^ tumors (PDAC^low^: meanC_I _= 738 ± 250 µg/g; *p* = 0.01). Corresponding relationships were discovered for Gd (PDAC^high^: meanC_Gd _= 27 ± 12 µg/g versus PDAC^low^: meanC_Gd _= 146 ± 45 µg/g; *p* = 0.008). To further support the interchangeability of CE-µCT and DCE-MRI, MSI-derived concentrations of I and Gd were correlated with AUC₆₀ values of tumor (*n* = 10) and muscle (*n* = 2) ROIs. As expected, tissue concentrations of both elements correlated strongly with AUC₆₀ as shown for I (Fig. 4c, *r* = 0.82; 95% CI = 0.46–0.95; *p* = 0.001) and Gd (Fig. 4d, *r* = 0.73; 95% CI = 0.26–0.92; *p* = 0.009).

## Discussion

In this pilot study we employ a murine endogenous and highly heterogeneous model of pancreatic cancer to compare the tissue regional discriminatory power for tumor subregions and the intermodal comparability of CE-µCT- and DCE-MRI-derived imaging parameters. We perform in vivo and ex vivo correlations of imaging-derived mean regional biomarkers HU and AUC₆₀ and MSI-derived CA concentrations. Semi-quantitative analyses of regional HU and AUC₆₀ show excellent distinction of histologically defined tumor subregions of low versus high tumor cellularity (*p* < 0.0001 for both). Furthermore, we noted a strong intermodal correlation between mean regional HU and AUC₆₀ values (*r* = 0.91, 95% CI = 0.78–0.97), as well as between mean regional I and Gd ion concentrations (*r* = 0.86, 95% CI = 0.55–0.96).

Imaging-derived biomarkers have been proposed for tumor characterization in PDAC ^3, 4, 5, 6^. For example, CE-CT-based patient studies have shown significantly higher contrast‑agent accumulation in PDAC regions with low tumor cellularity ^5, 6^ elevated stroma cell and extracellular matrix content 4, and better vascularization compared with tumors exhibiting high cellularity ^4, 6, 7^. Importantly, our imaging focuses on the initial 60-second phase of contrast agent distribution, which is largely determined by vascular density, early perfusion, and extravasation, rather than delayed retention or clearance ^8, 42^. In this context, PDAC^low^ tumors, with their dense stroma, provide a larger extracellular space that can accommodate contrast agents, resulting in more rapid early CA accumulation. Conversely, PDAC^high^ tumors, which are more densely cellular and have less extracellular matrix, show slower initial CA uptake, as the limited vascular and extracellular space restricts immediate distribution of low-molecular-weight contrast agents. These dynamics align with the principle that early-phase uptake is driven by accessible extracellular volume and vascular surface area. This interpretation is supported by prior PDAC studies showing that tumor mechanics, stromal abundance, and tumor cellularity modulate vascular access, while elevated interstitial pressure further influences the spatial distribution of solutes within the tumor microenvironment ^6, 43, 44^. Moreover, the non-invasive characterization of m/hPDAC tumor composition using initial mean CA accumulation detected by CE-CT and DCE-MRI is of high clinical importance, because high tumor cellularity was correlated to aggressive PDAC subtype, the presence of metastases in solid organs and shorter recurrence-free and overall survival in patients ^3,4,25^.

In addition, CE-CT and DCE-MRI derived parameters have been used for pre-therapeutic tumor stratification and treatment monitoring in PDAC. Here, low intratumoral CA accumulation at baseline in CE-CT prior FOLFIRINOX treatment predicted shorter patient survival and detected highly aggressive squamous PDAC subtype ^7^. In our previous study, high CA accumulation observed in baseline CE-CT of patients and DCE-MRI of animals, especially in stroma-rich PDAC^low^ tumors, was correlated to significantly diminished perfusion under Gemcitabine treatment ^6^. These imaging observations were also associated to lower endothelial proliferation and reduced chemotherapy accumulation caused by Gemcitabine. Furthermore, Cao and colleagues showed that DCE-MRI in mice can detect alteration in stromal composition caused by hyaluronan degradation via PEGPH20 (pegvorhyaluronidase alfa) in PDAC ^33^. Since most PDAC tissues contain high amounts of complex, dynamic stroma that impairs vascular function and drug delivery ^43^, non-invasive in vivo characterization of PDAC tumors is of interest and could serve as an imaging-derived biomarker in clinical trials. However, the targeted treatment of stromal compartment has failed in clinical trials ^45^.

CE-µCT studies using clinically approved iodinated CAs such as iomeprol in murine PDAC are lacking. This is most likely explained by the poor soft tissue contrast of preclinical CE-µCT with iomeprol (Fig. 2a, Suppl. Fig. 1) compared to DCE-MRI with gadopentetate dimeglumine^6,33,46,47^. In this study, CE-µCT alone provided limited anatomical information, so accurate tumor delineation required co-registration with T2-weighted MRI, highlighting the value of multi-modal imaging in preclinical studies. However, with a special focus on regional tumor uptake, we detect a differential CA accumulation pattern of murine PDAC^low^ and PDAC^high^ in both CE-µCT and DCE-MRI in vivo. Mean regional CE-µCT and DCE-MRI enhancement values within the initial 63 (CE-µCT) or 60 (DCE-MRI) seconds showed an excellent correlation within the same animal (Fig. 2b) enabling distinction of lesions of high and low tumor cellularity and confirming an inverse association of CA enhancement and tumor cellularity in µCT (Fig. 2c). The strong correlations between µCT‑ and MRI‑derived metrics observed in this pilot study likely reflect that I- and Gd-concentrations at the imaged time points fell within ranges in which both modalities exhibit limited or comparable nonlinear behavior. X-ray attenuation remains approximately proportional to I-concentration, and MRI signal enhancement is largely linear with Gd until much higher concentrations introduce relaxation saturation or T2 effects. Staying below these thresholds likely contributed to the close agreement observed between the two methods. The slight difference in acquisition duration between CT and DCE‑MRI is negligible compared with the fundamental methodological differences between the modalities and their sensitivity to rapid temporal changes in contrast uptake. While µCT requires a ~ 63‑second projection‑based acquisition that can integrate evolving contrast concentrations into a single static image, the much shorter 0.6‑second DCE‑MRI frames minimize temporal blurring and allow the first‑minute signal to be averaged as an AUC measure, making the comparison robust despite the differing acquisition schemes. In addition, mean regional gadopentetate dimeglumine and iomeprol CA concentrations showed a strong positive correlation (*r* = 0.86, Fig. 3c) measured ex vivo, confirming in vivo observations. Moreover, similar accumulation patterns (Fig. 4a, b) were observed in regions of low and high tumor cellularity for concentrations of I and Gd via LA-ICP-MS.

Few prior studies in humans compared the diagnostic power of both CE-CT and DCE-MRI within the same patient. In a study that investigated CA accumulation in human liver metastases a high correlation was reported for AUC₆₀ and CT-derived parameters ^32^. Another study compared PDAC DCE-MRI and CE-CT in PDAC patients ^6^ in combination with histopathological analyses of tumor cellularity and found a similar differential CA accumulation patterns for PDAC^low^ and PDAC^high^ lesions, as well as hypovascularity of PDAC^high^ for both modalities ^6^. In addition, a review study comparing performance of MRI and CT in the presurgical evaluation of pancreatic cancer revealed a high similarity grade for both methods^48^. Importantly, our study does not address clinical tumor delineation performance, but rather evaluates whether contrast-agent distribution patterns observed in preclinical DCE-MRI are concordant with those measured by µCT, thereby providing a necessary translational link between widely used preclinical MRI biomarkers and clinically dominant CT-based imaging of PDAC.

There are limitations of this pilot study. Our findings are based on a small number of animals, which limits its statistical power and generalizability. In addition ROI-based analyses in animal no. 1 with CE-µCT and DCE-MRI were performed using manual delineation on T2-weighted MRI with subsequent transfer to CE-µCT, which introduces a degree of observer dependence and potential variability in ROI placement despite the use of standardized anatomical landmarks and consensus review. Fully automated or deformable multimodal registration was not feasible due to differences in imaging platforms and acquisition conditions. However, our prior work on a large cohort of mice (56 tumor ROIs derived from 46 animals) also found the distinction of PDAC^low^ and PDAC^high^ by DCE-MRI feasible ^6^. Although the small sample size in this study precludes definitive conclusions about population-level effects, our findings—particularly the LA-ICP analysis (Fig. 3 and Suppl. Fig. 2) - provide a solid foundation for future studies with larger cohorts and support the feasibility of correlating CE-µCT and DCE-MRI signals with intratumoral heterogeneity in murine PDAC. In addition, the imaging signal in MRI and CT depends on technical parameters in CT (e.g. tube voltage) and MRI (e.g. flip angle and pre-scan adjustments) and may even vary across different CA concentrations. However, a thorough investigation of these variations is beyond the scope of this work.

In conclusion, this study demonstrates similar contrast‑agent accumulation patterns for iomeprol and gadopentetate dimeglumine in vivo using CE‑µCT and DCE‑MRI, as well as comparable iodine and gadolinium tissue‑distribution profiles in murine PDAC ex vivo. These findings underscore the translational value of both imaging modalities for non-invasive evaluation of tumor biology and therapeutic response, which is clinically highly relevant given the association of low vascularity with aggressive disease and poor prognosis.

## Supplementary Information

Below is the link to the electronic supplementary material.

Supplementary Material 1

## Data Availability

The datasets generated and analysed during the current study are available from the corresponding author on reasonable request.
