# Supplementary material for: Contrast enhanced CT and MRI interchangeably reflect tumor characteristics in murine pancreatic cancer
Source: Sci Rep. 2026 Jun 5;16:17516. doi: 10.1038/s41598-026-56008-4 (PMC13241501; doi:10.1038/s41598-026-56008-4)

Supplementary Information for the manuscript:

Contrast enhanced CT and MRI interchangeably reflect tumor characteristics in murine pancreatic cancer

Lea Würfel[1*], Peter Niehaus[2], Geoffrey J. Topping[3], Mariia Semina[1], Markus Mittelhäuser[3], Susanne Kossatz[3], Marina Lesina[5], Fabian Lohöfer[1], Franz Schilling[3,4], Uwe Karst[2], Rickmer Braren[1,4,6+*] and Irina Heid[1+*]

Suppl. Figure 1: Exemplary slide co-registration of T2-weighted MRI and CE-μCT images acquired within the first 63 seconds, illustrating high tumor heterogeneity (top) and manual ROI placement (bottom) in both modalities for this animal. Coronal views were selected to maximize the number of displayed ROIs.

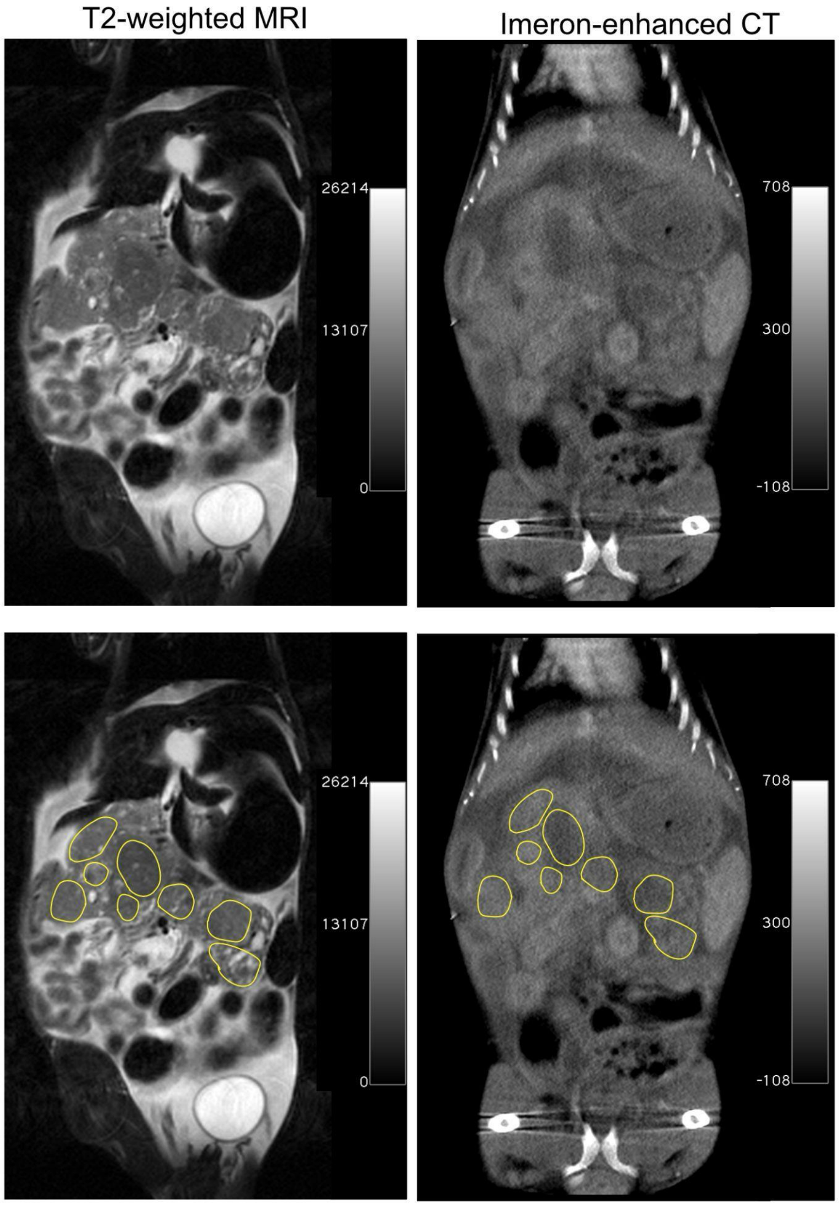

Suppl. Figure 2: Additional examples of ex vivo quantification of CA uptake in mPDAC. Left: H&E-stained overview of subregions (scale bar = 1 mm). Middle/right: Ex vivo LA-ICP-MS–derived elemental distribution maps of I and Gd corresponding to the H&E images. Regions of interest (ROIs) are color-coded, with black indicating mPDAC^{low} and red indicating mPDAC^{high} regions. For animal 1, tissue ROIs correspond to CE-µCT and DCE-MRI images shown in Fig. 2; for animal 2 (upper row), ROIs correspond to the well-perfused tumor shown on the left in Fig. 1.

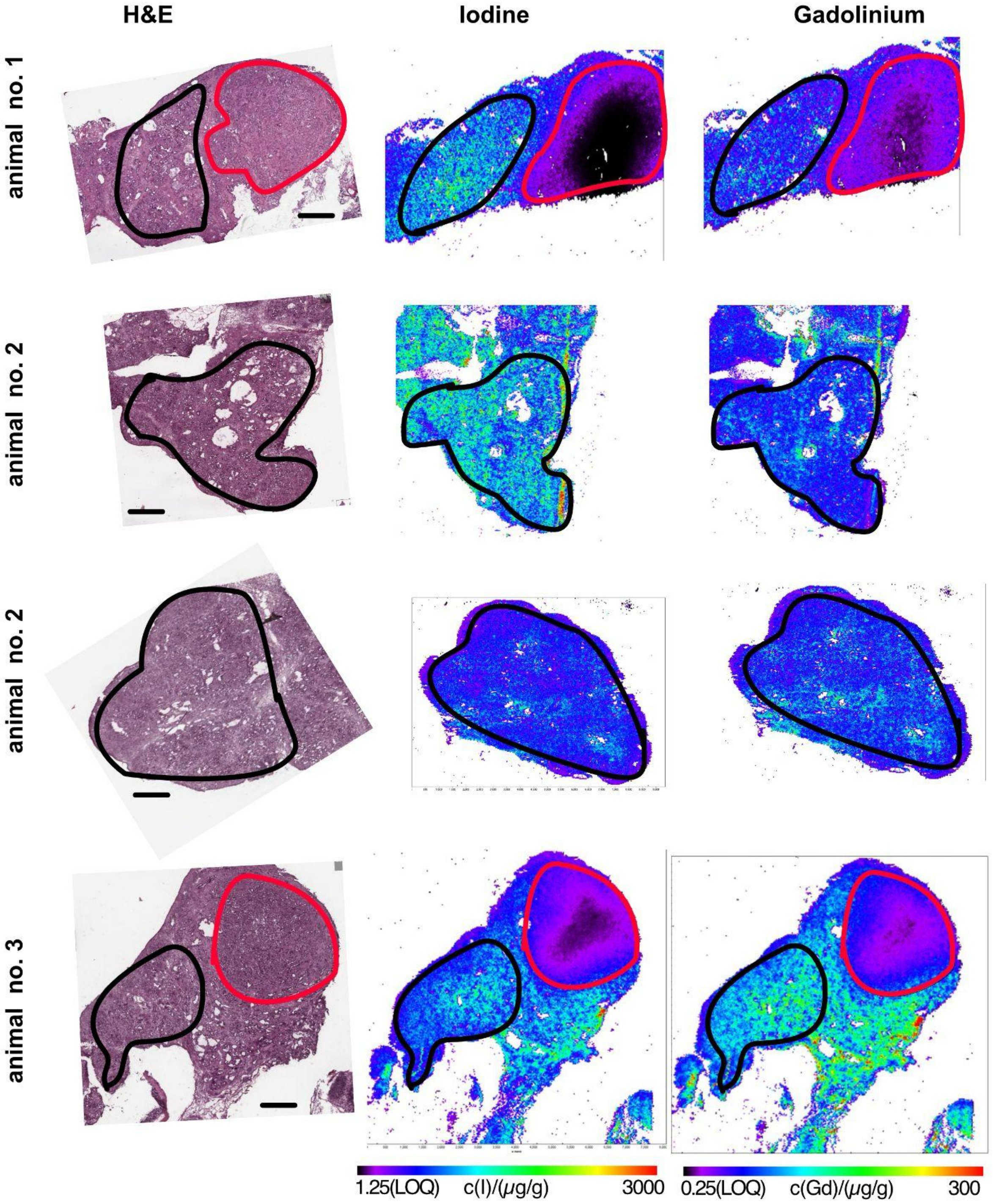

Supplement: Supplementary file 1 — Supplementary Material 1 [file 41598_2026_56008_MOESM1_ESM.pdf]
